# Surface and underwater human pose recognition based on temporal 3D point cloud deep learning

**DOI:** 10.1038/s41598-023-50658-4

**Published:** 2024-01-02

**Authors:** Haijian Wang, Zhenyu Wu, Xuemei Zhao

**Affiliations:** 1 School of Mechanical and Electrical Engineering, Guilin University of Electronic Technology, Guilin, 541004 Guangxi China; 2 School of Electronic Engineering and Automation, Guilin University of Electronic Technology, Guilin, 541004 Guangxi China

**Keywords:** Computer science, Electrical and electronic engineering

## Abstract

Airborne surface and underwater human pose recognition are crucial for various safety and surveillance applications, including the detection of individuals in distress or drowning situations. However, airborne optical cameras struggle to achieve simultaneous imaging of the surface and underwater because of limitations imposed by visible-light wavelengths. To address this problem, this study proposes the use of light detection and ranging (LiDAR) to simultaneously detect humans on the surface and underwater, whereby human poses are recognized using a neural network designed for irregular data. First, a temporal point-cloud dataset was constructed for surface and underwater human pose recognition to enhance the recognition of comparable movements. Subsequently, radius outlier removal (ROR) and statistical outlier removal (SOR) were employed to alleviate the impact of noise and outliers in the constructed dataset. Finally, different combinations of secondary sampling methods and sample sizes were tested to improve recognition accuracy using PointNet++. The experimental results show that the highest recognition accuracy reached 97.5012%, demonstrating the effectiveness of the proposed human pose detection and recognition method.

## Introduction

Drowning is the third leading cause of unintentional injury deaths worldwide and one of the top ten major causes of death among children and young adults^1^. One of the primary factors contributing to drowning incidents is the lack of lifeguards or insufficient supervision by lifeguards in most open-water areas. However, because of the vast expanse of water bodies globally, it is not feasible to assign lifeguards to supervise them all. Therefore, the development of an automated monitoring system for drowning prevention is urgently required. Surface and underwater human pose recognition is key to the development of such an automated monitoring system.

Researchers have conducted a series of studies on the recognition of surface and underwater human poses, which are divided into two main categories. The first method involves monitoring drowning incidents through wearable sensors, which require individuals to wear sensor devices. Monitoring of signals such as pressure^2^, heart rate^3^, acceleration^4^, inertia^5^, and position^6^ can indicate a drowning situation. However, this method is costly and can restrict the movements of swimmers because of the requirement to wear sensor devices, potentially leading to drowning incidents, which does not align with the original research intention. Considering these issues, methods based on image or video recognition have been proposed, including background subtraction^7^, hue saturation value (HSV)^8^, k-means clustering algorithm^9^, and deep learning^10–12^. While recognizing postures through videos or images resolves the issues associated with wearable sensors, the use of airborne optical cameras poses challenges in simultaneously imaging both the water surface and underwater because of the influence of visible light wavelengths. Furthermore, the lack of spatial geometric information in images can lead to misjudgment and misdetection of poses.

LiDAR technology has demonstrated advantages in addressing the challenges of image-based recognition of water surfaces and underwater human poses. LiDAR possesses strong penetration capabilities because laser beams can penetrate the water surface and generate dense 3D point clouds, providing detailed 3D information about the water surface and underwater human poses. Unlike RGB images, 3D point clouds contain both geometric and spatial information. To fully utilize this information, Li et al.^13^ first conducted research on human pose recognition using depth image sequences. This approach involves sampling points from the depth image and encoding the obtained point-cloud model to extract spatial information regarding the human pose. However, this method of computing normal vectors and constructing feature descriptors in the spatiotemporal domain relies on predefined shape descriptors or manually extracted features, and often fails to fully express the complexity and richness of point cloud data.

Compared to traditional machine learning methods, deep learning approaches are better suited for handling 3D point-cloud data and possess stronger feature learning and expression capabilities. In the context of deep-learning-based methods for 3D point clouds, researchers have utilized the point cloud data generated by millimeter-wave radar, along with information such as distance, Doppler, and micro-Doppler, to extract features related to human poses. They employ deep learning neural networks, such as convolutional neural networks (CNN)^14^, long short-term memory (LSTM)^15^, and 3D residual network (Res3D)^16^, and further utilize voxelization to address the sparsity and nonuniformity of point cloud data, to research human pose recognition. However, because of the sparsity of the point-cloud data generated by LiDAR, where data samples are far fewer than the number of voxels, LiDAR point-cloud data voxelization significantly increases the system memory and computational requirements, resulting in a large number of ineffective convolution calculations.

With the introduction of PointNet and PointNet++ 3D point cloud deep learning models by Qi et al.^17,18^ in 2016 and 2017, respectively, a new and effective approach emerged to address the issues associated with voxelization-based deep learning networks and point cloud disorder in 3D point cloud classification. Researchers^19,20^ have utilized the PointNet++ 3D point cloud deep learning method to improve the accuracy of human pose recognition through model enhancement, achieving higher recognition accuracy, which validates the advantages of the PointNet++ network in human pose recognition. However, all studies on PointNet++-based human pose 3D point cloud deep learning have focused on optimizing the network model to improve accuracy while neglecting the processing and optimization of the dataset. Most studies employing a single filtering method utilize the default furthest point sampling by PointNet++ to sample only once, setting the sample size to 1024 or 2048. The choice of the filtering method for water surface and underwater human-pose 3D point clouds necessitates secondary sampling for the methods involved, which needs to be investigated and explored along with the optimal sample size for achieving the highest recognition accuracy.

To address the existing problems and limitations in current research, an experimental simulation platform was established in chapter 2. In chapter 3, three-dimensional point clouds of human body model postures were collected and different methods were applied to preprocess the point clouds. Finally, after temporalizing the point clouds, model training was performed to obtain highly accurate results. Analyzes and compares the accuracy of various methods in chapter 4. This study investigated human pose recognition on water surfaces and underwater environments using the PointNet++ spatiotemporal 3D point-cloud deep learning method. A spatiotemporal point-cloud dataset was constructed to identify human poses in water and underwater scenarios. Subsequently, a series of comparative experiments were designed based on the dataset. The study explored two filtering methods to determine the optimal one, analyzed the necessity of incorporating secondary sampling for the methods involved, and investigated the influence of the sample size on classification accuracy. Finally, from the combinations of the two filtering methods, two secondary sampling methods and five sample sizes were selected, and the combination with the highest accuracy constituted the optimal method for surface and underwater human poses. We have constructed a time point cloud dataset for water surface and underwater human pose recognition, and obtained the optimal combination of methods for water surface and underwater human pose recognition. The highest classification accuracy achieved using PointNet++ is 0.975012. The solutions to these problems provide important guidance for the application of the PointNet++ spatiotemporal 3D point-cloud deep learning method.

## Experimental platform setup

### Experimental equipment

**Figure 1:**
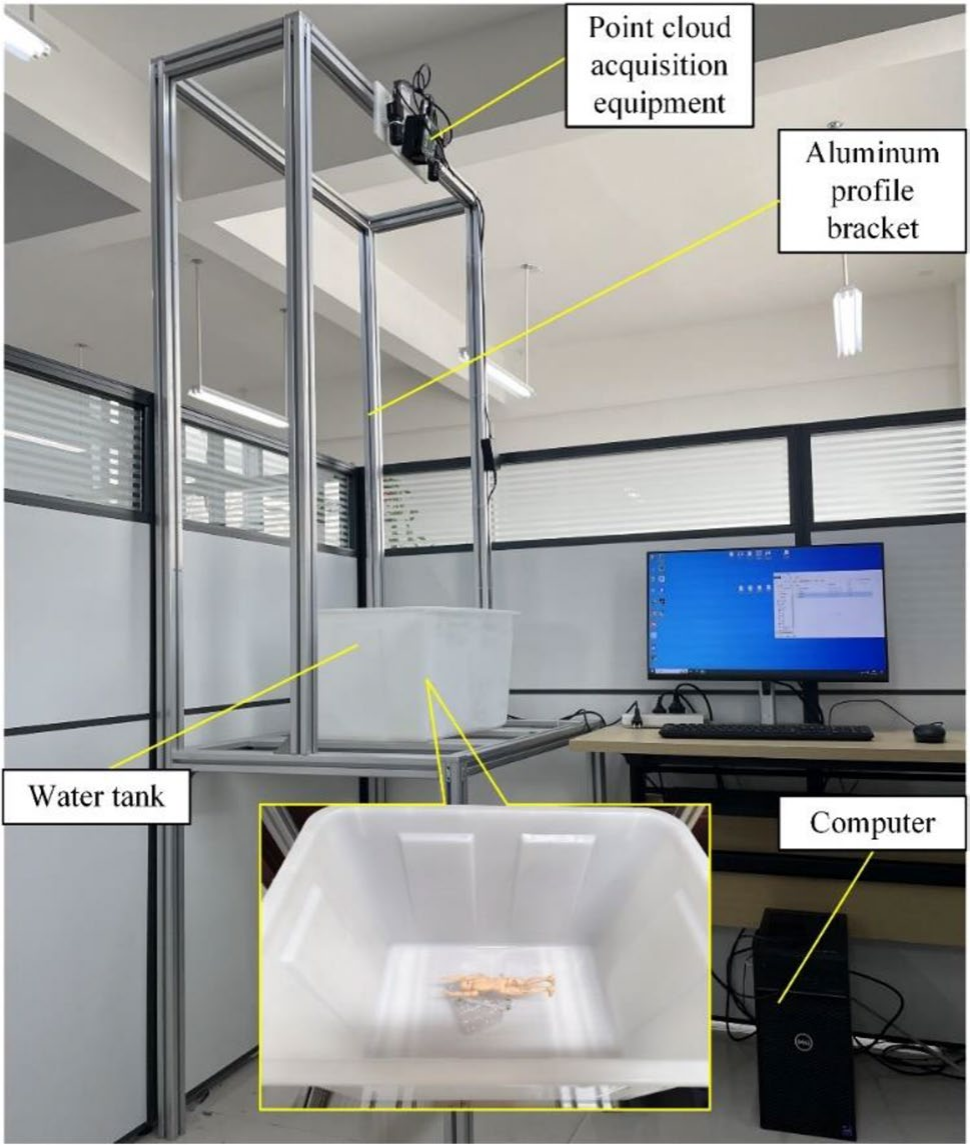
Experimental setup.

The experimental setup, as shown in Fig. 1, included a point cloud acquisition device, an aluminum frame support, a water tank, and a computer. The point cloud acquisition device utilizes a structured light binocular acquisition system, with an effective acquisition distance of approximately 1–1.5 m and a field of view of approximately 0.8 * 0.8 m^2^. The aluminum frame support was constructed using 4040 aluminum profiles. The size of the water tank was 0.51 * 0.38 * 0.29 m^3^. A computer with an i9-11900K @ 3.50 GHz processor and an RTX 3060 graphics card was used.

### Experimental objects

**Figure 2:**
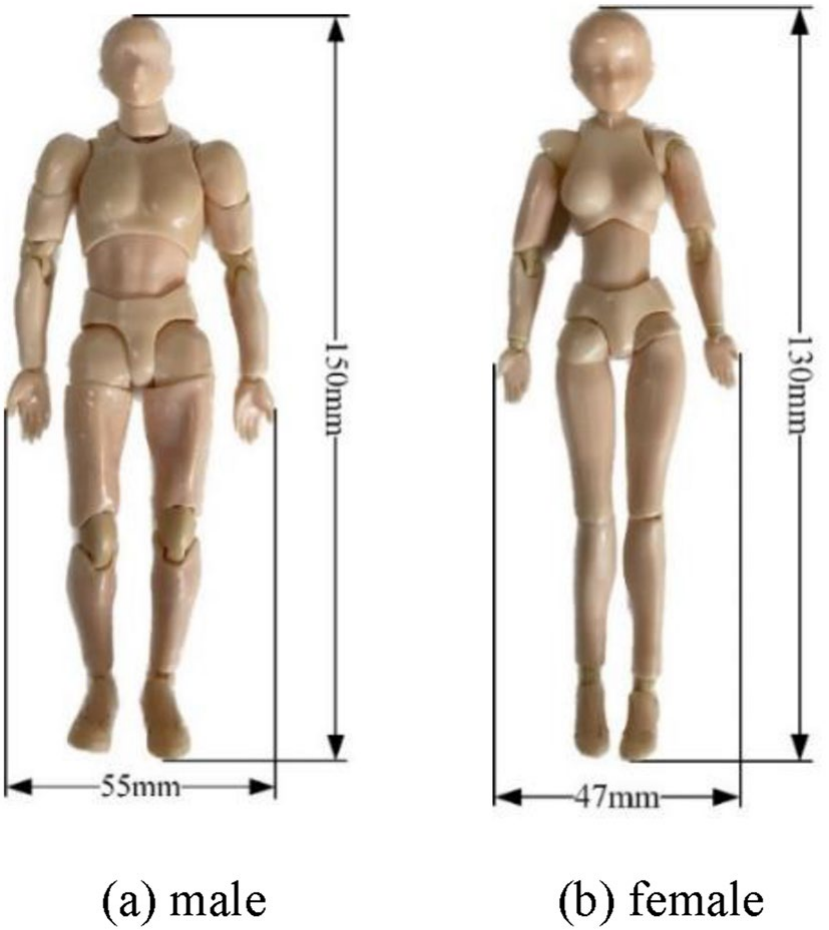
Human models.

The experimental equipment was used to scan objects, thereby collecting two male and female human models of approximately 1:12 scale. The water surface and underwater postures of the two human models were collected with a collection volume of 2400 for each model. The height of the male body model was approximately 150 mm and that of the female body model was approximately 130 mm, as shown in Fig. 2.

## Research proposal

**Figure 3:**
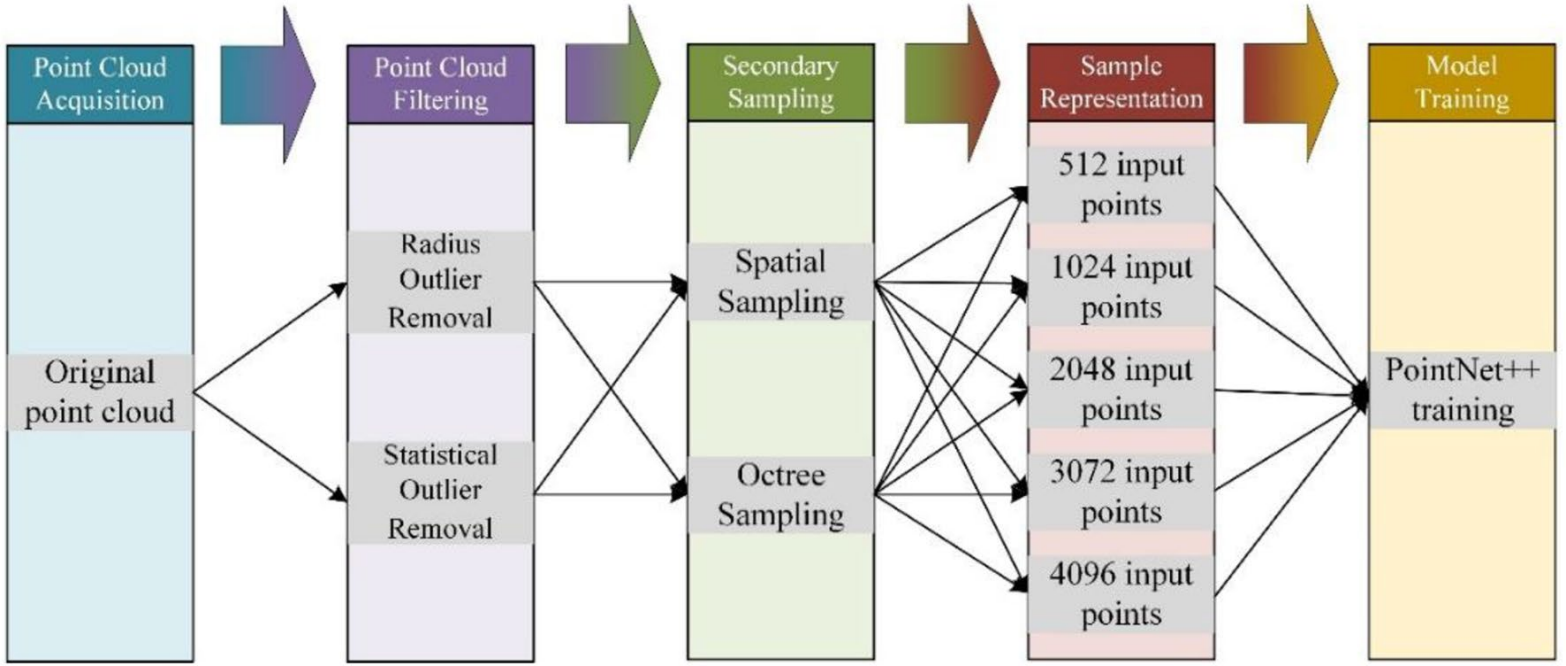
Flowchart.

To maximally utilize the information within the dataset while minimizing the impact of noise, this study proposes the flowchart shown in Fig. 3, which includes point cloud acquisition, point cloud filtering, point cloud secondary sampling, point cloud input, and model training. Point cloud preprocessing comprises point cloud filtering, point cloud secondary sampling, and sample representation. Point cloud filtering was used to filter the noise and outliers introduced during data collection. Secondary sampling with appropriate sample size was performed to further decrease the impact of noise and outliers. Finally, PointNet++ was utilized to recognize the continuity of the swimming actions.

### Point cloud acquisition

**Figure 4:**
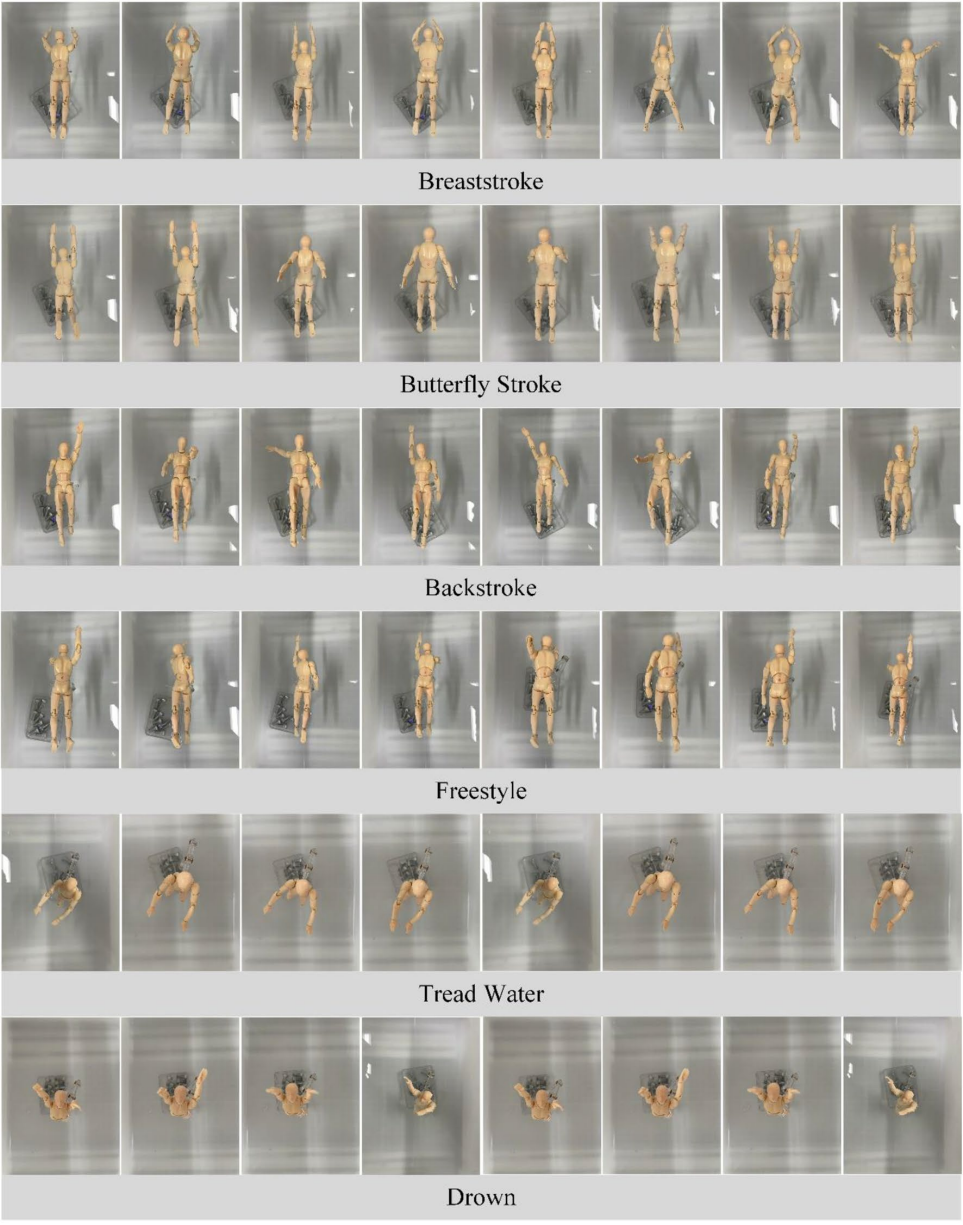
Human poses.

To simulate human pose while swimming, a point-cloud dataset was acquired using human models. This dataset included four common swimming postures: breaststroke, butterfly stroke, backstroke, and freestyle, as well as tread water, and drown postures. Considering the continuity of motion in these six poses, the complete process of the six poses was divided into eight segments on average based on time. The middle-frame pose of each segment is shown in Fig. 4.

**Figure 5:**
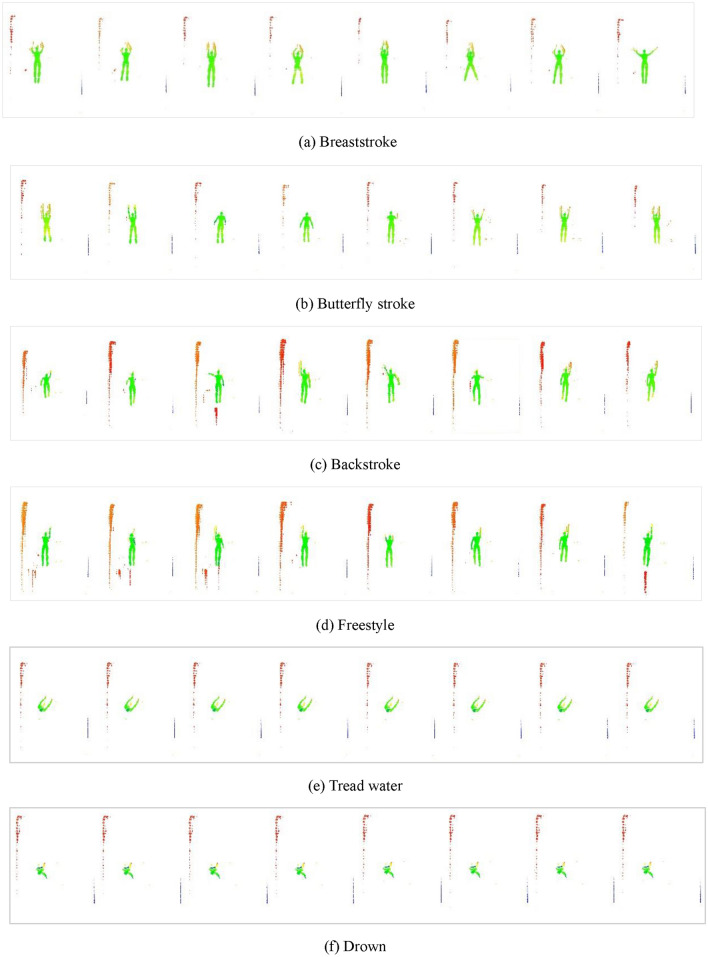
Original point clouds of human poses.

Through LiDAR scanning of surface and underwater human models, a total of 400 point cloud samples were obtained for each of the four swimming styles, tread water, and drowning. This enabled us to create a well-balanced training dataset. To better capture the swimmer’s continuous movements over time, each pose was divided into an average of eight actions and sampled 50 times to simulate various swimming poses. The corresponding information is summarized in Table 1. Typical samples for each middle-frame pose are shown in Fig. 5.

**Table 1:** Sample data quantification.

Pose	Number of decomposition actions	Number of point clouds per action	Total number of point clouds
Breaststroke	8	50	400
Butterfly stroke	8	50	400
Backstroke	8	50	400
Freestyle	8	50	400
Tread water	8	50	400
Drown	8	50	400

**Figure 6:**
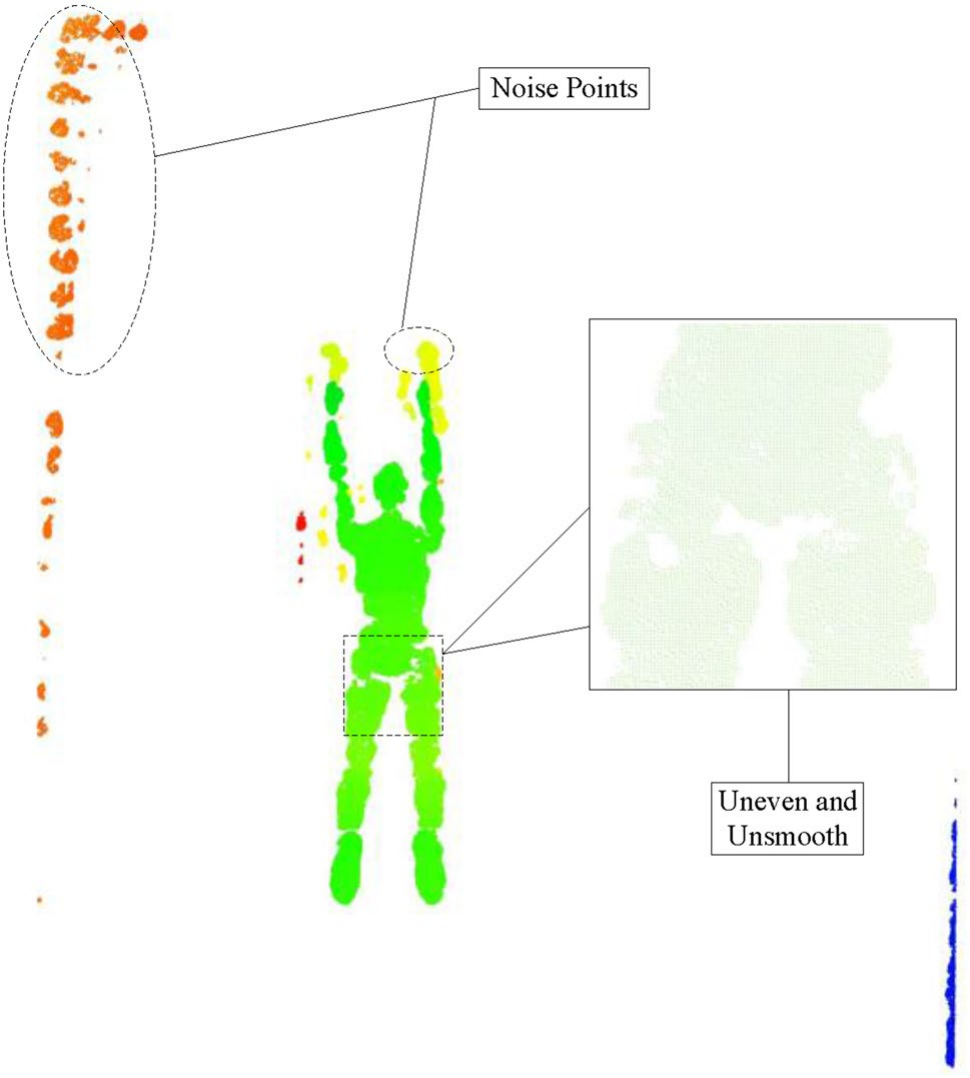
Characteristics of original point cloud data.

As shown in Fig. 6, the original point clouds contain noisy points that do not belong to the human model, such as water surfaces and water tanks. In addition, the raw point cloud has an uneven density and unsmooth edges (such as the part in the rectangular dashed box). These drawbacks affect the subsequent human pose recognition in the following process.

### Point cloud filtering

**Figure 7:**
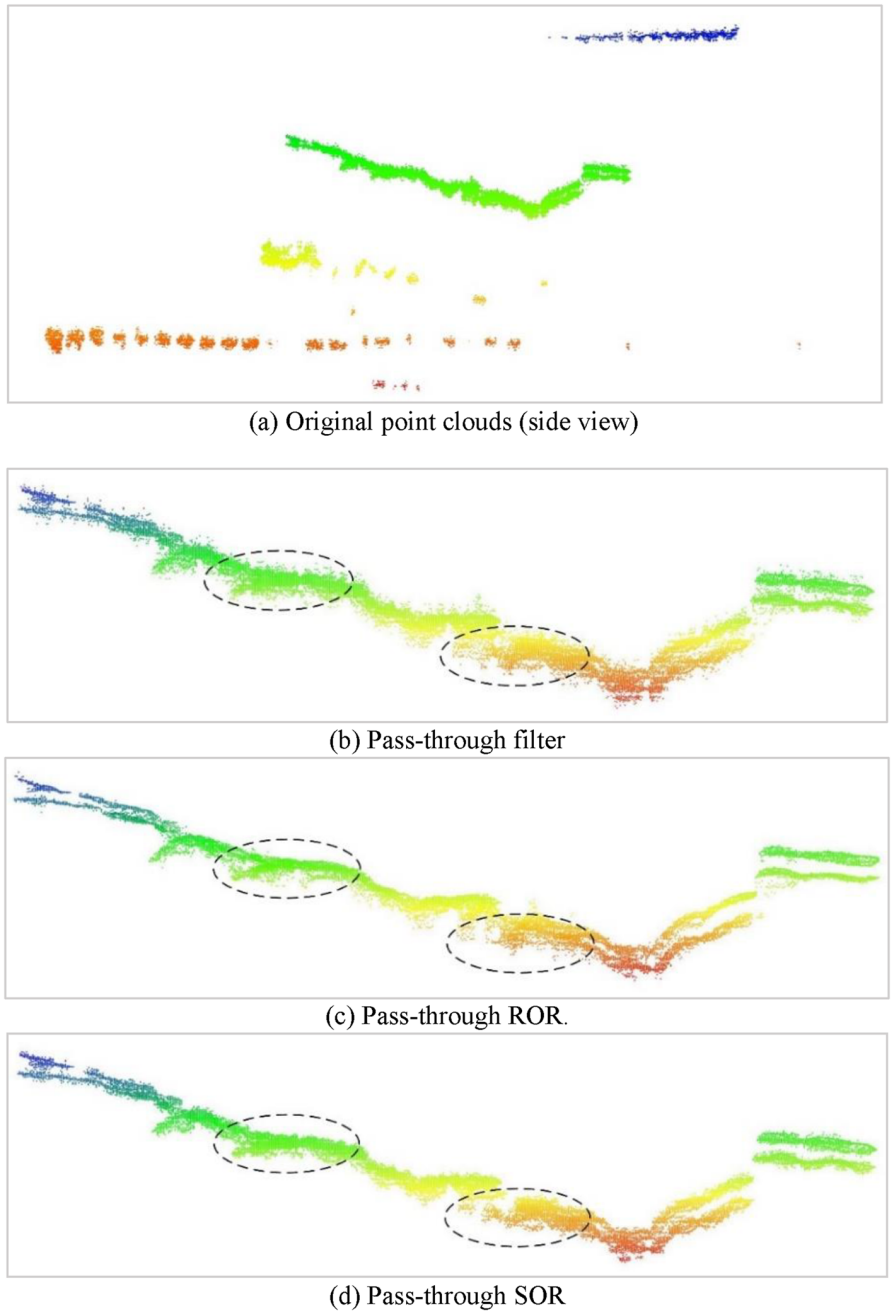
Point cloud preprocessing.

The original point clouds are shown in Fig. 7a. To alleviate the impact of noise outliers (indicated within the elliptical dashed box in Fig. 6), this study first utilized a pass-through filtering method^21^ that employs a z-coordinate threshold to roughly identify data points representing the human model. The threshold of the z-coordinates was set within the range of 1150–1200 mm, as shown in Fig. 7b, to filter out most of the irrelevant noise; however, noise remains near the human model. Accordingly, ROR^22^ and SOR^23^ were selected to remove this noise. The filtering results are shown in Fig. 7c,d, using a 3 mm radius and a threshold of 6 for the ROR and a neighborhood size of 50 points and a standard deviation multiplier of 1.0 for SOR. Comparing these results, the ROR retains more details, whereas the SOR tends to filter out more data points that may not be all noise. The point counts before and after preprocessing are listed in Table 2.

**Table 2:** Point count before and after preprocessing.

Pose	Original quantity	Passthrough filtering	Passthrough-ROR	Passthrough-SOR
Breaststroke	35,172	27,018	19,386	23,084
Butterfly Stroke	36,386	28,755	21,264	24,297
Backstroke	35,629	27,521	20,157	23,186
Freestyle	37,505	29,156	22,405	25,771
Tread Water	18,351	10,917	7745	9418
Drown	13,049	6952	6083	6692

### Point cloud secondary sampling

**Figure 8:**
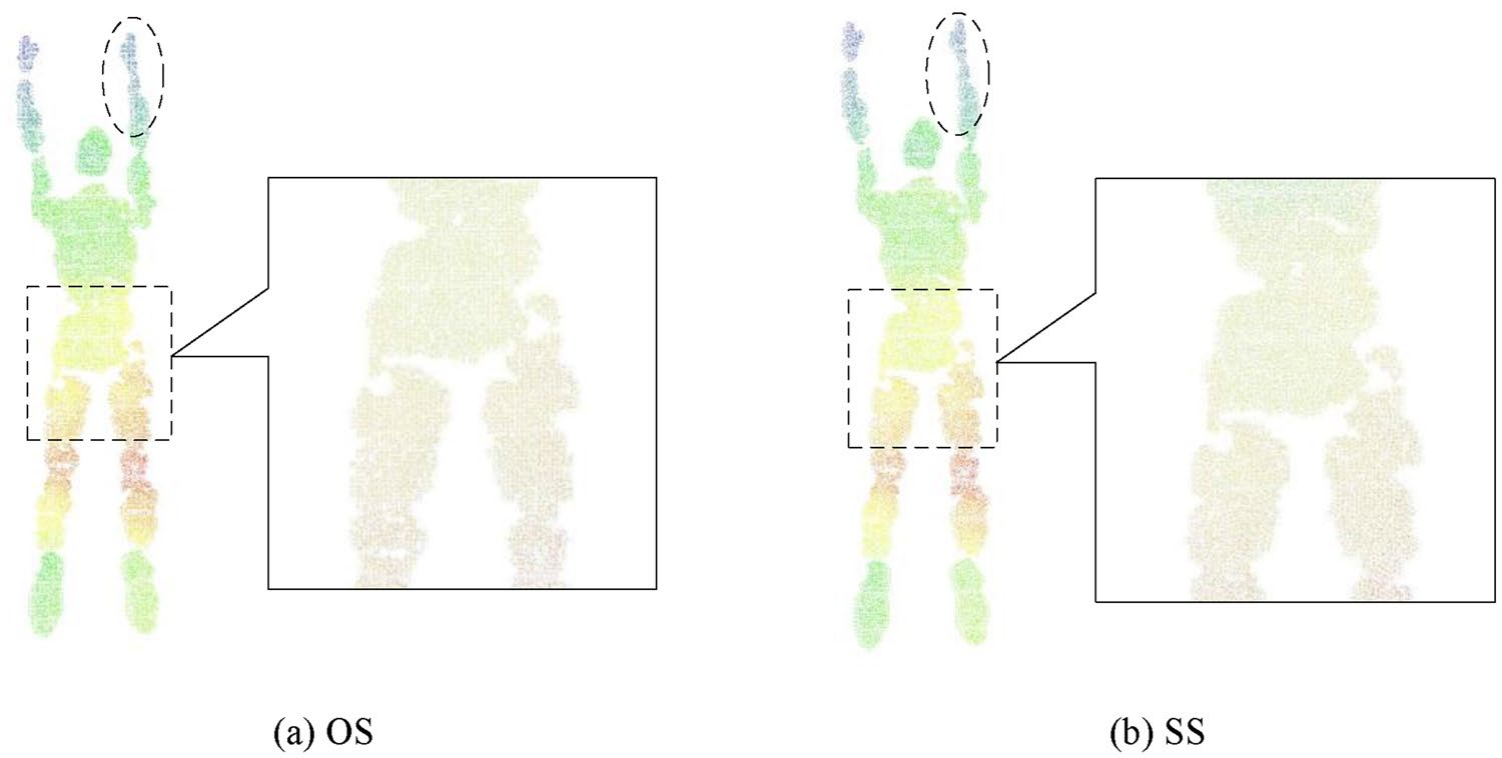
Point cloud secondary sampling.

In this study, PointNet++^18^ was employed to recognize human poses because of its exceptional performance in point-cloud processing. Before feeding the samples into PointNet++, the farthest point sampling (FPS)^24^ operator was employed to sample points that effectively represent human poses, to ensure uniform input data size. However, the improved filtered point cloud still exhibits some residual noise that may adversely affect the extraction of geometrical structures during FPS, particularly along the edges of human models. Therefore, we incorporated a secondary point-cloud sampling process to address this issue. The effects of spatial sampling (SS)^25^ and octree sampling (OS)^26^, using the data after pass-through SOR, are shown in Fig. 8. The SS method was set with a minimum point spacing threshold of 0.5 mm, and the OS method was set with a maximum recursion depth of 8. Comparing Fig. 8a,b, the point cloud density is more uniform with OS, obtaining slightly inferior results with SS. As it is not possible to determine the optimal sampling method directly, the effects of both sampling methods were verified through combined experiments.

### Sample representation

**Figure 9:**
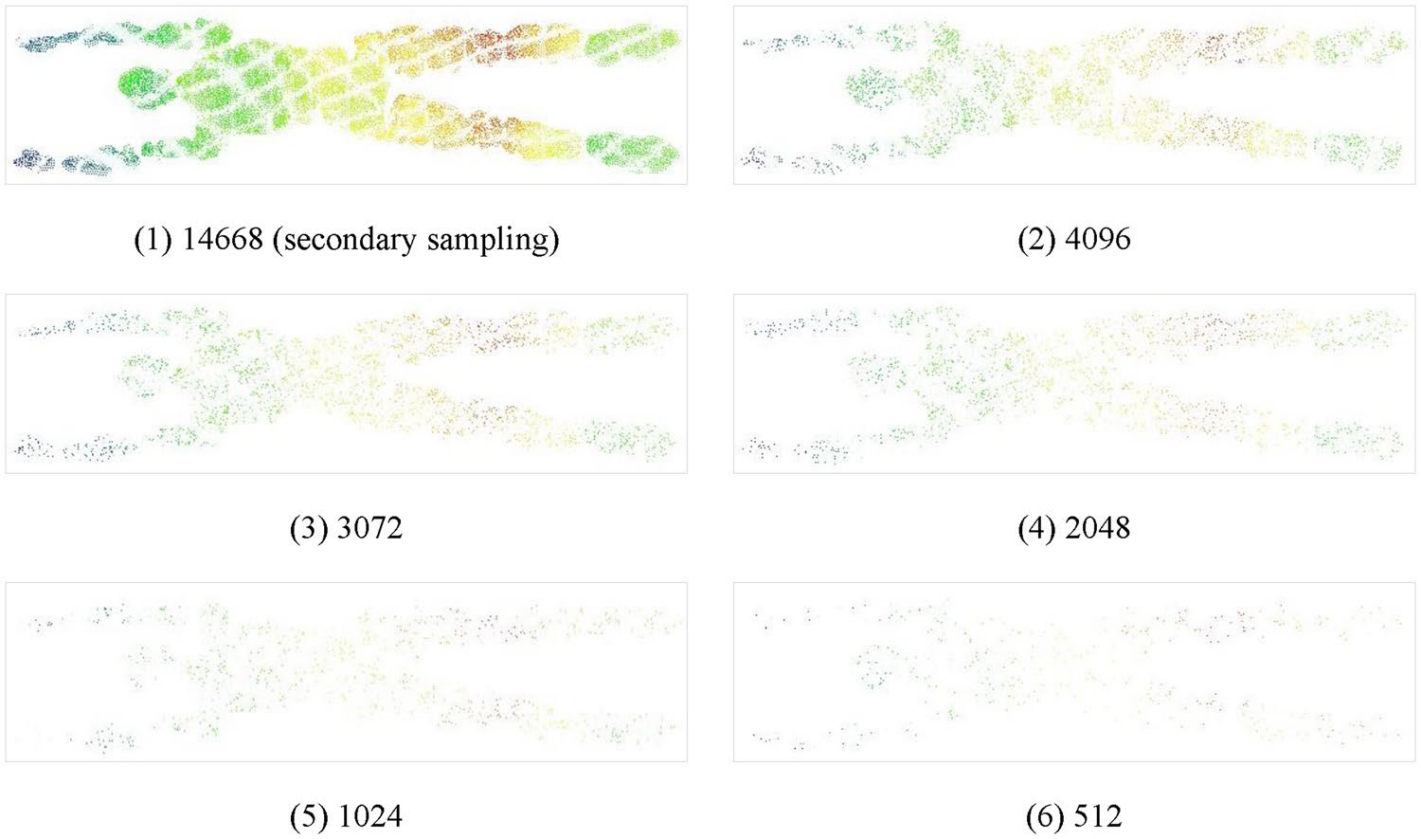
Sample representation.

The sample size fed into PointNet++ also had a significant impact on the learning ability of the network. The larger the sample size, the more information it contains but the greater is the computational load. To explore the optimal number of input points in surface and underwater human pose point-cloud classification, we chose five different sample sizes: 512, 1024, 2048, 3072, and 4096. As shown in Fig. 9, to fully utilize the continuity of motions representing the same pose, a variable representing the time series was added to the vector of each point. Thus, each point contained seven types of information: x, y, and z coordinates; x, y, and z normals; and the timestamp.

### Model training

To address the unordered nature of point clouds, Qi et al.^18^ proposed the PointNet++ three-dimensional point cloud deep learning model, whereby the local features of point clouds are utilized to significantly enhance recognition accuracy. Before training the PointNet++ model, the input data are normalized using the farthest point sampling method to avoid overfitting.

Unlike two-dimensional image deep learning models, the PointNet++ model solves two key issues in three-dimensional point cloud deep learning. The first concerns the unordered nature of point clouds and achieving permutation invariance for point clouds with inconsistent orders. The second issue is the large amount of point-cloud data and integration of local features to improve recognition accuracy.

To address the issue of unordered point clouds, PointNet++^18^ first uses *h* function to increase the dimensionality of the input data, then uses *g* function for max pooling, and finally uses the *γ* function for MLP operation. Map a set of points to a vector using the following formula:

1 $$ f\left( {x_{{1}} ,x_{{2}} , \ldots ,x_{n} } \right) = \gamma \left( {g\left( {h\left( {x_{{1}} } \right),h\left( {x_{{2}} } \right), \ldots ,h\left( {x_{n} } \right)} \right)} \right) $$

here* x*_1_,* x*_2_, …, *x*_*n*_ belong to various points in the point cloud; *f*(*x*_1_,* x*_2_, …, *x*_*n*_) represents the PointNet++ function; *γ* represents the MLP function about *g*; *g* represents the Max function about *h*; and *h* represents the dimensionality elevation function about *x*_1_,* x*_2_, …, *x*_*n*_.

**Figure 10:**
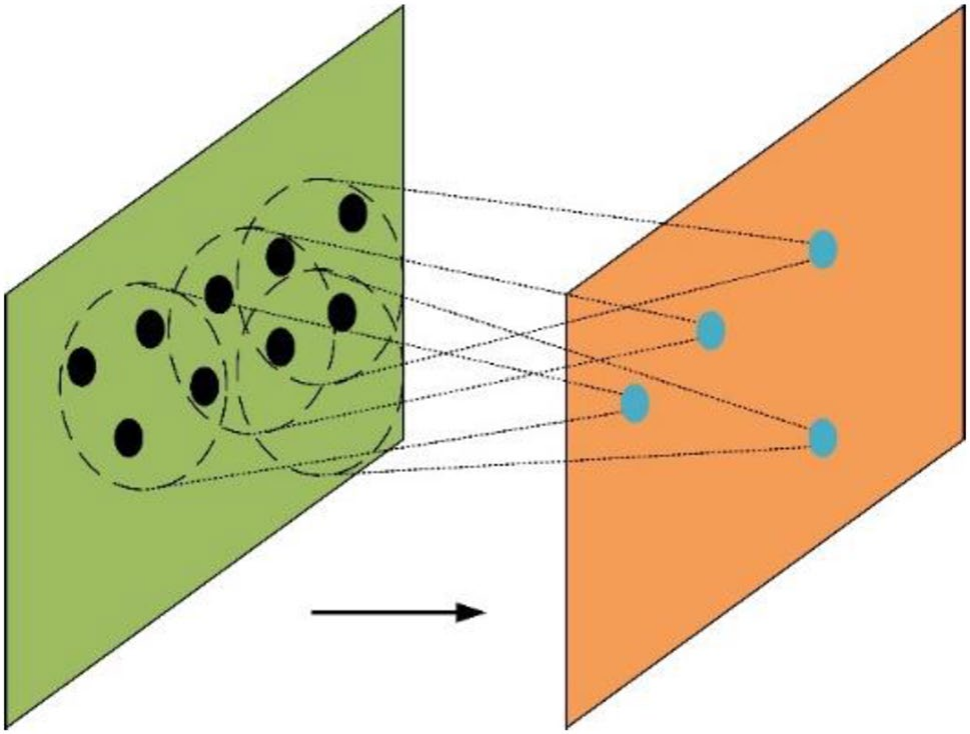
Fusion of local features.

To integrate local features, PointNet++ selects local regions based on a specified radius and performs feature extraction for each region. The local features are then combined to obtain the global features, as shown in Fig. 10.

A point-cloud dataset containing different combinations of time sequences was used to train the PointNet++ model. Table 3 presents the parameters used in model training. We randomly divided the dataset into training, validation, and testing sets in a ratio of 6:2:2 for PointNet++ network training.

**Table 3:** Model training.

Hyperparameter	Value
Model	pointnet2_cls_msg
Batch size	8
Decay rate	0.0001
Epoch	200
Learning rate	0.001
Sample Sizes	512, 1024, 2048, 3072, 4096
The number of categories of objects	6
Optimizer	Adam

## Results

To address the challenges of sample temporalization and manual selection of filters, samplers, and sample sizes, 80 experiments were designed to cover all possible combinations, as shown in Tables 4 and 5, where A_1_ and A_2_ denote the filtering algorithms; B_1_ and B_2_ denote the secondary sampling algorithms; B_3_ denotes no secondary sampling; C_1_, C_2_, C_3_, C_4_ and C_5_ denote the sample sizes; D_1_ denotes no temporalization; D_2_ denotes temporalization; E_1_ denotes male body models; and E_2_ denotes female body models. Among them, 20 experiments (a combination of two filtering algorithms, five sample sizes, and two body models without secondary sampling and temporalization) represented the non-temporalized traditional recognition method based on PointNet++. Another 20 experiments (a combination of two filtering algorithms, five sample sizes, one temporalization, and two body models without secondary sampling) introduced temporalization into the traditional method, representing the temporalized traditional recognition method. The remaining 40 experiments (a combination of two filtering algorithms, two secondary sampling algorithms, five sample sizes, one temporalization, and two body models) incorporated point-cloud secondary sampling and temporalization into the traditional method, representing the temporalized secondary sampling recognition method. The effectiveness of sample temporalization and secondary sampling for point-cloud human pose recognition was validated by comparing the results of these 80 experiments. The study also identified the filtering algorithms with the highest classification accuracy among the two choices and explored the optimal combination of filtering methods, secondary sampling methods, and sample sizes for underwater human pose recognition based on temporal 3D point cloud deep learning.

**Table 4:** Experimental variables.

Serial number	Experimental variables	Code	Serial number	Experimental variables	Code
1	ROR	A_1_	8	2048 points	C_3_
2	SOR	A_2_	9	1024 points	C_4_
3	OS	B_1_	10	512 points	C_5_
4	SS	B_2_	11	No temporalized	D_1_
5	No secondary sampling	B_3_	12	Temporalized	D_2_
6	4096 points	C_1_	13	Male	E_1_
7	3072 points	C_2_	14	Female	E_2_

**Table 5:** Experimental combination.

Serial number	Experimental combination	Serial number	Experimental combination	Serial number	Experimental combination
1	A_1_B_3_C_1_D_1_E_1_	28	A_1_B_3_C_4_D_2_E_2_	55	A_1_B_2_C_3_D_1_E_1_
2	A_1_B_3_C_1_D_1_E_2_	29	A_1_B_3_C_5_D_2_E_1_	56	A_1_B_2_C_3_D_1_E_2_
3	A_1_B_3_C_2_D_1_E_1_	30	A_1_B_3_C_5_D_2_E_2_	57	A_1_B_2_C_4_D_1_E_1_
4	A_1_B_3_C_2_D_1_E_2_	31	A_2_B_3_C_1_D_2_E_1_	58	A_1_B_2_C_4_D_1_E_2_
5	A_1_B_3_C_3_D_1_E_1_	32	A_2_B_3_C_1_D_2_E_2_	59	A_1_B_2_C_5_D_1_E_1_
6	A_1_B_3_C_3_D_1_E_2_	33	A_2_B_3_C_2_D_2_E_1_	60	A_1_B_2_C_5_D_1_E_2_
7	A_1_B_3_C_4_D_1_E_1_	34	A_2_B_3_C_2_D_2_E_2_	61	A_2_B_1_C_1_D_1_E_1_
8	A_1_B_3_C_4_D_1_E_2_	35	A_2_B_3_C_3_D_2_E_1_	62	A_2_B_1_C_1_D_1_E_2_
9	A_1_B_3_C_5_D_1_E_1_	36	A_2_B_3_C_3_D_2_E_2_	63	A_2_B_1_C_2_D_1_E_1_
10	A_1_B_3_C_5_D_1_E_2_	37	A_2_B_3_C_4_D_2_E_1_	64	A_2_B_1_C_2_D_1_E_2_
11	A_2_B_3_C_1_D_1_E_1_	38	A_2_B_3_C_4_D_2_E_2_	65	A_2_B_1_C_3_D_1_E_1_
12	A_2_B_3_C_1_D_1_E_2_	39	A_2_B_3_C_5_D_2_E_1_	66	A_2_B_1_C_3_D_1_E_2_
13	A_2_B_3_C_2_D_1_E_1_	40	A_2_B_3_C_5_D_2_E_2_	67	A_2_B_1_C_4_D_1_E_1_
14	A_2_B_3_C_2_D_1_E_2_	41	A_1_B_1_C_1_D_1_E_1_	68	A_2_B_1_C_4_D_1_E_2_
15	A_2_B_3_C_3_D_1_E_1_	42	A_1_B_1_C_1_D_1_E_2_	69	A_2_B_1_C_5_D_1_E_1_
16	A_2_B_3_C_3_D_1_E_2_	43	A_1_B_1_C_2_D_1_E_1_	70	A_2_B_1_C_5_D_1_E_2_
17	A_2_B_3_C_4_D_1_E_1_	44	A_1_B_1_C_2_D_1_E_2_	71	A_2_B_2_C_1_D_1_E_1_
18	A_2_B_3_C_4_D_1_E_2_	45	A_1_B_1_C_3_D_1_E_1_	72	A_2_B_2_C_1_D_1_E_2_
19	A_2_B_3_C_5_D_1_E_1_	46	A_1_B_1_C_3_D_1_E_2_	73	A_2_B_2_C_2_D_1_E_1_
20	A_2_B_3_C_5_D_1_E_2_	47	A_1_B_1_C_4_D_1_E_1_	74	A_2_B_2_C_2_D_1_E_2_
21	A_1_B_3_C_1_D_2_E_1_	48	A_1_B_1_C_4_D_1_E_2_	75	A_2_B_2_C_3_D_1_E_1_
22	A_1_B_3_C_1_D_2_E_2_	49	A_1_B_1_C_5_D_1_E_1_	76	A_2_B_2_C_3_D_1_E_2_
23	A_1_B_3_C_2_D_2_E_1_	50	A_1_B_1_C_5_D_1_E_2_	77	A_2_B_2_C_4_D_1_E_1_
24	A_1_B_3_C_2_D_2_E_2_	51	A_1_B_2_C_1_D_1_E_1_	78	A_2_B_2_C_4_D_1_E_2_
25	A_1_B_3_C_3_D_2_E_1_	52	A_1_B_2_C_1_D_1_E_2_	79	A_2_B_2_C_5_D_1_E_1_
26	A_1_B_3_C_3_D_2_E_2_	53	A_1_B_2_C_2_D_1_E_1_	80	A_2_B_2_C_5_D_1_E_2_
27	A_1_B_3_C_4_D_2_E_1_	54	A_1_B_2_C_2_D_1_E_2_	–	–

### Impact of sample temporalization on model classification accuracy

**Figure 11:**
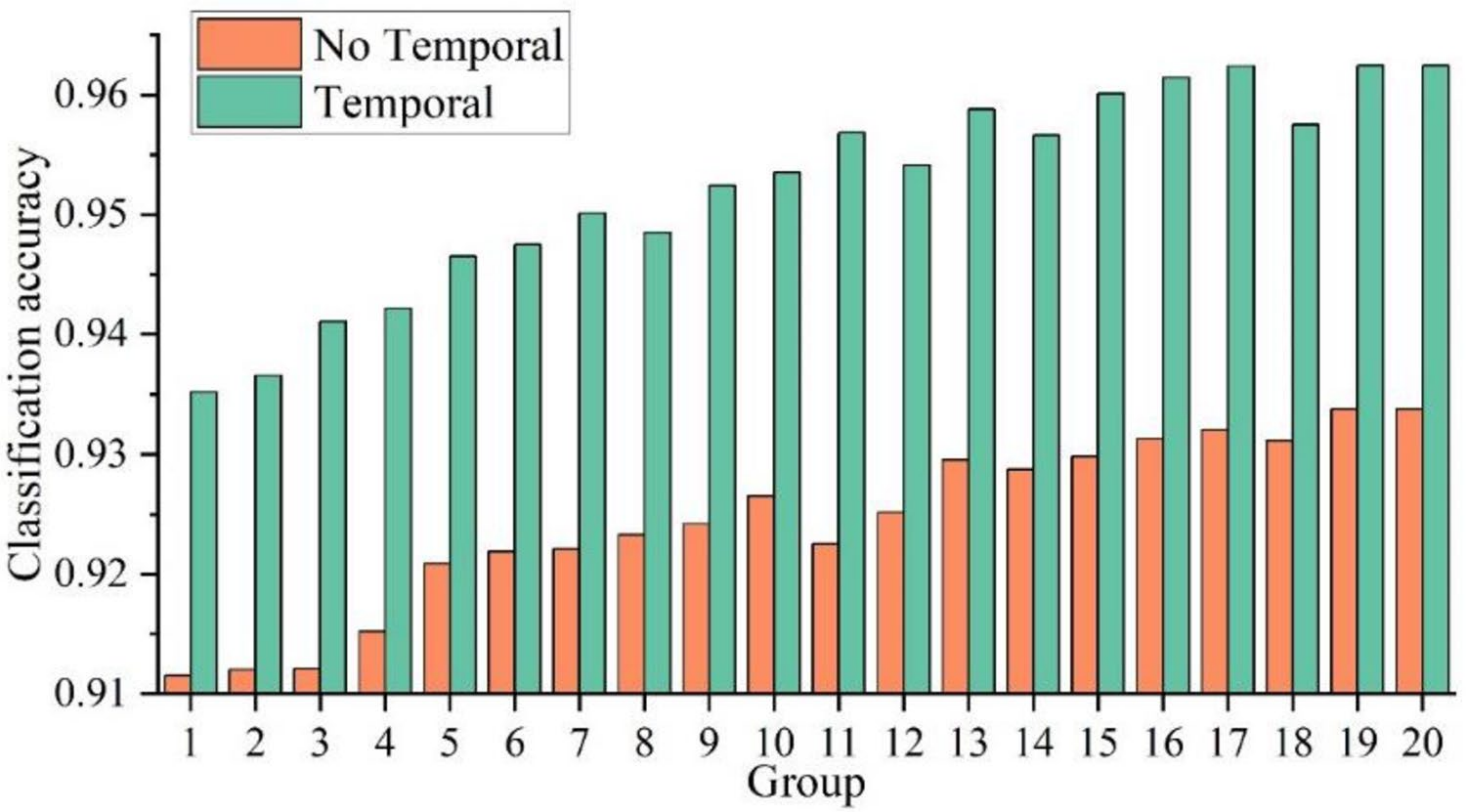
Comparison of model classification accuracy before and after sample temporalization.

The effect of sample temporalization on the classification accuracy of traditional recognition models was verified through 20 sets of comparative experiments based on PointNet++. These experiments included 20 (a combination of two filtering algorithms, five sample sizes, and two body models without secondary sampling and temporalization) experiments representing the non-temporalized traditional recognition method. An additional 20 (a combination of two filtering algorithms, five sample sizes, one temporalization, and two body models without secondary sampling) experiments represented the temporalized traditional recognition method. A comparison of the model classification accuracies before and after sample temporalization is shown in Fig. 11. In the 20 sets of comparative experiments, the classification accuracy of all the models with sample temporalization was higher than those without sample temporalization, with the largest difference in accuracy being 0.034331. The average classification accuracy of the models without sample temporalization was 0.924003, whereas that of the models with sample temporalization was 0.952465, resulting in an average accuracy improvement of 0.028462. Thus, sample temporalization improves the accuracy of surface and underwater human pose recognition. This is because human poses are composed of continuous actions, and non-temporalized samples can result in the misclassification of similar actions.

### Impact of point cloud secondary sampling on model classification accuracy

**Figure 12:**
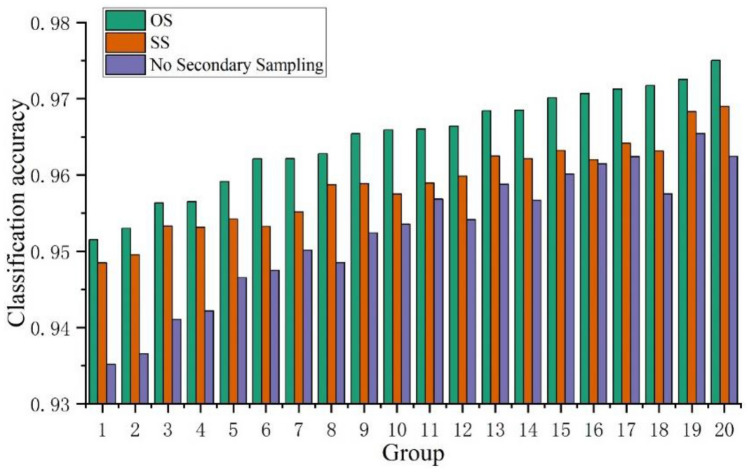
Comparison of model classification accuracy before and after point cloud secondary sampling.

The reasonableness of the proposed point-cloud secondary sampling process for recognizing drowning based on 3D point-cloud deep learning was validated through 20 sets of comparative experiments. These experiments included 20 (a combination of two filtering algorithms, five sample sizes, one temporalization, and two body models without secondary sampling) experiments representing the temporalized traditional recognition method and 40 (a combination of two filtering algorithms, two secondary sampling algorithms, five sample sizes, one temporalization, and two body models) experiments representing the temporalized secondary sampling recognition method. A comparison of the model classification accuracy before and after point cloud secondary sampling is shown in Fig. 12. In the 20 sets of comparative experiments, all models incorporating point-cloud secondary sampling had higher classification accuracies than those without point-cloud secondary sampling, with the largest difference in accuracy being 0.016437. The average classification accuracy of models without point cloud secondary sampling was 0.952465, whereas the average classification accuracy of models using SS as the secondary sampling method was 0.964782, and the average classification accuracy of models using OS as the secondary sampling method was 0.958761. The average classification accuracy using SS as the secondary sampling method was 0.012317 higher than that of the models without point-cloud secondary sampling and 0.006022 higher than that of the models using OS. Based on the results, incorporating secondary sampling is beneficial for improving model accuracy. This is because secondary sampling helps address the issue of uneven sample collection, and the use of OS has a better effect in this regard.

### Influence of different filtering methods on model classification accuracy

**Figure 13:**
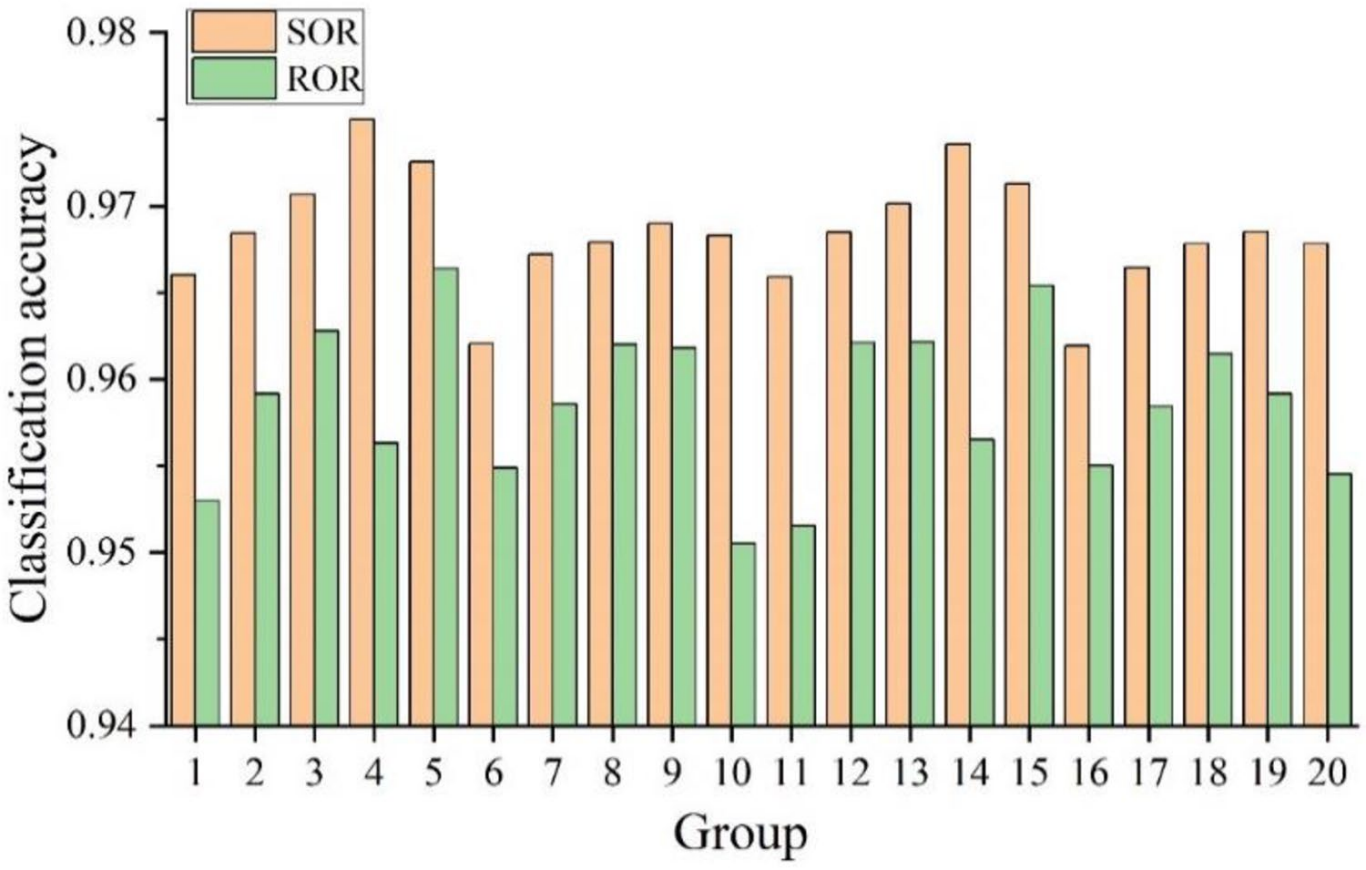
Comparison of model classification accuracy for two filtering methods.

To determine the best filtering method for surface and underwater human-body pose recognition based on 3D point-cloud deep learning, we conducted 20 sets of comparative experiments. These included 40 experiments (a combination of two filtering algorithms, two secondary sampling algorithms, five sample sizes, one temporalization, and two body models), representing the temporalized secondary sampling recognition method, as shown in Fig. 13. In all 40 experiments, the classification accuracy exceeded 0.940000. The highest classification accuracy of 0.975012 was achieved using SOR. In the 20 sets of comparative experiments, the classification accuracy of all the SOR experiments was higher than that of the ROR experiments. From an overall perspective, SOR performs better than ROR because it maintains the statistical distribution of the point cloud. By contrast, ROR can only eliminate visually noticeable independent noise.

### Impact of different method combinations on model classification accuracy

**Figure 14:**
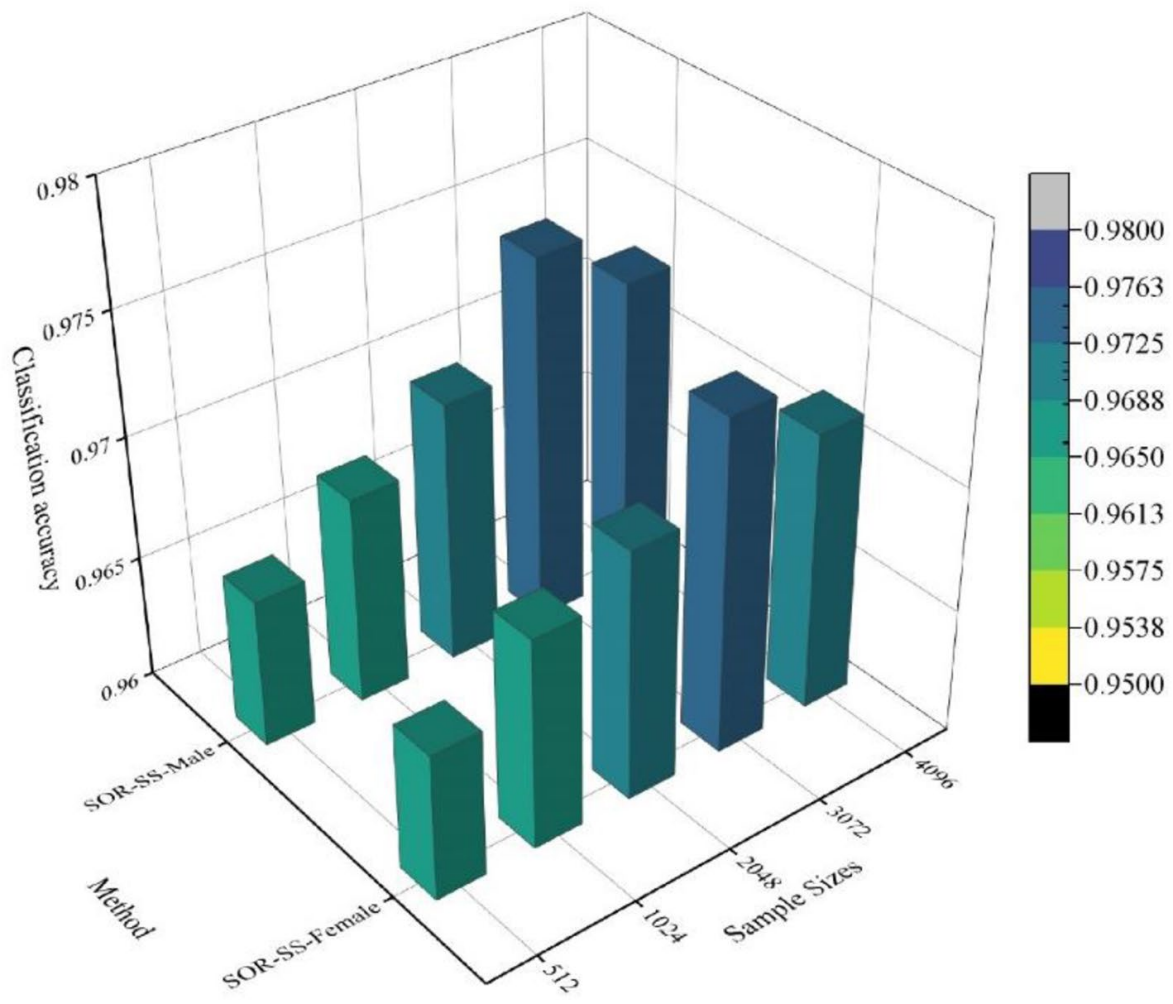
Comparison of model classification accuracy for different method combinations.

To explore the optimal combination of preprocessing methods, point cloud secondary sampling methods, and the impact of sample point numbers on surface and underwater human pose recognition based on three-dimensional point cloud deep learning, we conducted 10 experiments based on SOR-SS (a combination of one filtering algorithm, one secondary sampling algorithm, five sample sizes, one temporalization, and two body models). A comparison of the model classification accuracies for different combinations of the methods is shown in Fig. 14. The figure shows that the highest model classification accuracy was achieved with SOR, SS, and 3072 sample points. The classification accuracies of the male and female body models are 0.975012 and 0.973571, respectively. The lowest model classification accuracy was obtained with SOR, OS, and 512 sample points, with classification accuracies of 0.961652 and 0.961548 for the male and female body models, respectively. Therefore, it can be inferred that using SOR, SS, and a sample size of 3072 can improve model accuracy. This is because the sample size determines the amount of information contained. However, because of the suboptimal nature of manually collected point clouds, excessively large sample sizes can introduce irrelevant information, thereby reducing model accuracy.

### Comparison of classification accuracy with other point cloud recognition networks

Our dataset was trained in the same experimental environment, using several popular point-cloud recognition networks. A computer with an i9-11900K @ 3.50 GHz processor and an RTX 3060 graphics card was used. The experimental results are listed in Table 6. The PointNet++ network used in this study has higher classification accuracy than the other networks.

**Table 6:** Comparison of classification accuracy.

Point cloud recognition networks	Classification accuracy
3D-CNNs^27^	0.949452
Voxel-based networks^28^	0.955173
Sparse convolutional networks^29^	0.952415
PointNet^17^	0.960544
PointNet++	0.975012

## Conclusion

The results show that the proposed LiDAR method can be effectively employed for surface and underwater human pose recognition using temporal 3D point cloud deep learning.

1. The impact of sample temporalization on model classification accuracy was investigated, and the average accuracy increased by 0.028462 after sample temporalization. This is advantageous in improving the recognition ability of surface and underwater human-body pose recognition models.
2. The necessity of incorporating point-cloud secondary sampling was discussed. The classification accuracy of the surface and underwater human body pose recognition models significantly improved after adding point-cloud secondary sampling. Among them, SS had the best effect, with an average increase of 0.012317 in accuracy.
3. The optimal point-cloud filtering algorithm for surface and underwater human pose recognition based on temporal 3D point-cloud deep learning was determined to be SOR. The experimental results showed that the classification accuracy using SOR was significantly higher than that using ROR, with an average precision increase of 0.009873.
4. The best combination of methods for surface and underwater human pose recognition was obtained based on temporal 3D point-cloud deep learning. When using SOR, the SS algorithm, and a sample input point size of 3072, the model achieved a significantly higher classification accuracy compared to the other combinations, achieving the highest classification accuracies for the male and female human models of 0.975012 and 0.973571, respectively.

Although this study demonstrated the potential of surface and underwater human pose recognition based on temporal 3D point-cloud deep learning, some limitations still need to be addressed. The experimental stage should be validated for practical applications. It is necessary to collect point-cloud data on the water surface and underwater human attitudes from real humans to train models and obtain more reliable recognition models. Furthermore, in practical applications, the swimming speeds of different individuals should be considered in selecting different point-cloud acquisition times for recognition.

## Data Availability

The dataset generated during the current study are not publicly available due production took a lot of time and money but are available from the corresponding author on reasonable request.
